# Metal Slot Color Filter Based on Thin Air Slots on Silver Block Array

**DOI:** 10.3390/nano9060912

**Published:** 2019-06-25

**Authors:** Youngsoo Kim, Kihwan Moon, Young Jin Lee, Seokhyeon Hong, Soon-Hong Kwon

**Affiliations:** Department of Physics, Chung-Ang University, Seoul 06974, Korea; youngsoo.kim94@gmail.com (Y.K.); sinbadra@gmail.com (K.M.); youngjin.lee.91@gmail.com (Y.J.L.); lechter@naver.com (S.H.)

**Keywords:** color filter, metal block, air slots, slot transmission

## Abstract

The human eye perceives the color of visible light depending on the spectrum of the incident light. Hence, the ability of color expression is very important in display devices. For practical applications, the transmitted color filter requires high transmittance and vivid colors, covering full standard default color spaces (sRGB). In this paper, we propose a color filter with a silver block array on a silica substrate structure with nanoscale air slots where strong transmission is observed through the slots between silver blocks. We investigated the transmitted color by simulating the transmission spectra as functions of various structure parameters. The proposed structure with an extremely small pixel size of less than 300 nm covers 90% of sRGB color depending on the structure and has a narrow angular distribution of transmitted light.

## 1. Introduction

Modern nanophotonic devices have been developed based on wavelength-sized or subwavelength-sized nanostructures, such as photonic crystals [1,2,3,4], surface plasmon polaritons (SPPs) [5,6], and metamaterials [7,8,9]. In particular, SPPs have been researched because of their unique ability to manipulate light in devices with subwavelength size.

Recently, researchers have focused on controlling the phase and amplitude of reflected or transmitted light in the visible range to develop nanophotonic devices such as color filters [10,11,12], lenses [13,14,15], and displays [12,16,17]. Recently, plasmonic color filters for image sensors have been intensively studied because of their unique spectral characteristics, such as multi-spectral bands from visible to near-infrared light [18] or high spectral resolution [19]. In particular, the key parameters of visible nanophotonic devices are the transmittance or reflectance spectrum, linewidth of the resonance, corresponding colors, and polarization dependencies.

The controlled (transmitted or reflected) light should have high intensity for practical applications. In addition, if the spectrum of the controlled light of an optical device has narrow linewidth, it can be expected to show vivid colors with a large color gamut of the visible light area and, therefore, color control devices such as color filters and displays should be applied. When linearly polarized light enters a narrow low index dielectric slot embedded in a high index dielectric or metal, the light confides strongly in the low index slot forming a slot waveguide mode [20,21,22]. The transmission spectrum through the slot can be controlled to the indices of materials and slot thickness [20,23,24]. In this paper, we called this phenomenon “slot transmission”. 

In this paper, we focused on air slots of size tens of nanometers in the metal to control the transmission colors of visible light by proposing a color filter with cross-patterned air slots embedded in silver blocks on a silica substrate with a sub-300 nm pixel size. We investigated the transmission color of the proposed structure by changing the height and width of blocks at a fixed slot thickness. We also discussed the angular distribution of the transmitted light by estimating the far-field patterns for the representative structures of red, green, and blue colors.

## 2. Structure Design

**Figure 1:**
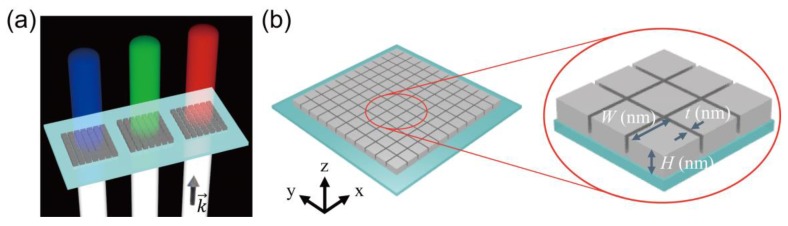
(**a**) Schematic diagram of proposed nanostructures, showing red, green, and blue transmissions; (**b**) Detailed view of the air slot structure embedded in silver blocks. The entire structure consists of silver blocks placed on silica substrate. Each silver block is defined by two parameters: width (*W*) and height (*H*). The slot thickness (*t*) is the size of air gap between two silver blocks.

We proposed a silver block array on the silica (SiO_2_) substrate structure as a color filter (Figure 1a), in which an ultra-thin air slot of thickness 20 nm (*t*) is sandwiched between rectangular parallelepiped silver blocks, as shown Figure 1b. The silver block is described by two parameters: width (*W*) and height (*H*). The structure is symmetric on the XY plane to suppress the polarization dependence on the incident light. It was assumed that linearly polarized light *E_x_* was normally incident from the bottom of the substrate, and the transmitted light represented the demanded color. The proposed structure was designed to act as a color filter in the visible light area by adjusting the width and height of the block to control the spectrum of the transmitted light.

In this paper, we used a homemade three-dimensional (3D) finite-difference time-domain (FDTD) method for simulations of the transmittance spectrum and far-field pattern of the proposed structure. The validation of the homemade code was tested for various nanophotonic structures, photonic crystals, nanoparticles, metamaterials, plasmonic structures, by comparison with a commercial FDTD code (Lumerical) and COMSOL finite element method (FEM) code. In addition, the experimentally measured optical properties of such structures agree well with the numerical results obtained from the homemade FDTD code [25,26,27,28]. All simulations were performed with a spatial resolution of 5 nm, and periodic boundary conditions on the XY plane and perfectly matched layer (PML) boundary condition on the z-axis were used. The refractive index of SiO_2_ is 1.47 and the optical constant of the silver was fitted using the Drude-critical point model based on the experimentally determined values [29].

First, in order to cover the entire visible color with changing structure parameters of the silver block, we investigated the transmittance spectra for different heights (*H*) and widths (*W*) of the silver block. In the simulations, the slot thickness (*t*) was fixed to 20 nm to confirm the dependencies on the width (*W*) and height (*H*) of the silver block. Air slot between silver blocks can be fabricated by focused ion beam (FIB) etching, as used in the previously reported SPP air slot waveguides [30,31] because of the fabrication flexibility of FIB method. On the other hand, the self-assembly of metal nanocubes can also be applied to fabricate the proposed structure because of the advantage of mass production if the difficulties to control gap size uniformly and make asymmetric metal blocks are solved [32,33]. 

**Figure 2:**
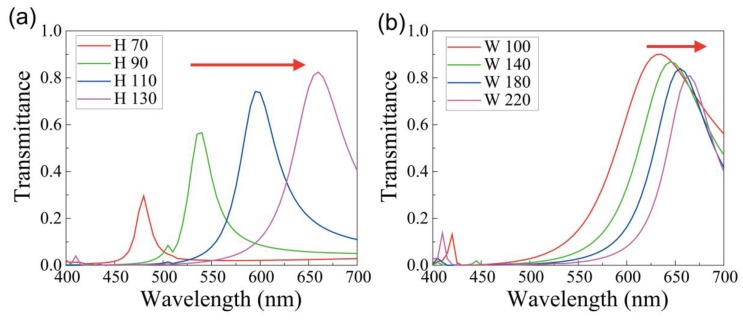
(**a**) Transmittance spectra at different heights (*H*) of the silver block: 70 nm (red), 90 nm (green), 110 nm (blue), and 130 nm (magenta). The width (*W*) and slot thickness (*t*) are fixed to 200 and 20 nm, respectively; (**b**) Transmittance spectra for different widths of the silver block: 100 nm (red), 140 nm (green), 180 nm (blue), and 220 nm (magenta). The height and slot thickness are fixed to 130 and 20 nm, respectively.

Figure 2 shows the transmittance spectra at different heights (*H*) and widths (*W*), where there is one dominant transmission peak. For the fixed width *W* = 200 nm and slot thickness *t* = 20 nm, as height increases from 70 to 130 nm, the peak transmittance increases from 0.3 to 0.8, and the peak red shifts from 480 to 670 nm (Figure 2a). When the transmission peak is placed in the short wavelength region of the blue color, the transmittance is relatively small as 0.3. Since a strong transmission is observed at the resonance of the plasmonic mode excited at the air slot [21,24], the wavelength and the transmittance of the resonance depend on the height of the slot. On the other hand, the transmittance spectra show small red-shifts of the peak for wavelengths ranging from 634 to 665 nm, and a slight decrease in the peak transmittance from 0.90 to 0.81 with an increase in the width of the block from 100 to 220 nm, as shown in Figure 2b. Here, height and slot thickness are fixed to 130 and 20 nm, respectively. As shown in Figure 2a,b, the strong dependence of the resonance wavelength on the height of the slot and weak dependence on the width of the block imply that the plasmonic mode mostly resonates the slot vertically.

## 3. Optimized Structure and Color Space

In the previous section, we investigated the changes in the transmittance spectrum by controlling the size of silver blocks. Based on the previous result shown in Figure 2, we calculated the colors of the transmitted light by converting the transmittance spectrum obtained from the finite-difference time-domain (FDTD) simulations depending on the changes in the width (*W*) and height (*H*) of silver blocks at a fixed slot thickness of *t* = 20 nm. The colors matched to the Commission Internationale de l’Eclairage (CIE) color spaces. The parameters of silver blocks were changed at 20 nm step to height (*H*) and width (*W*) that ranged from 50 to 250 nm and 100 to 300 nm, respectively. 

**Figure 3:**
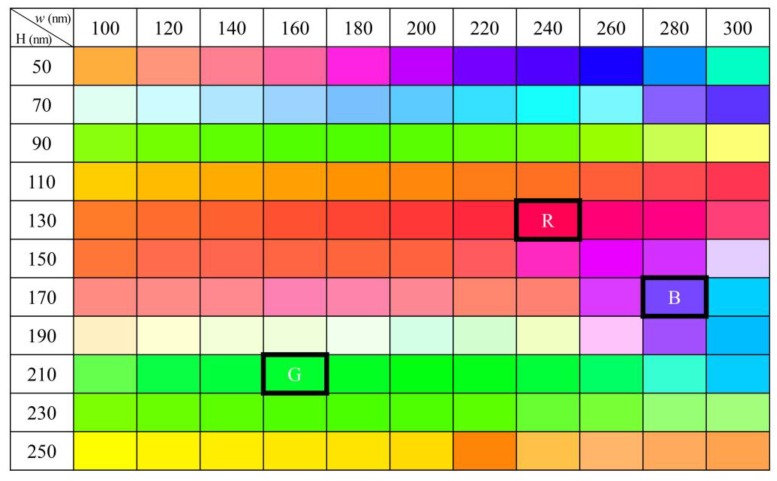
Color table of all cases simulated using the finite-difference time-domain (FDTD) method. The width (*W*) and height (*H*) of silver blocks range from 100 to 300 nm and from 50 to 250 nm, respectively. Three black outlined boxes indicate three representative structures and the corresponding colors, red (R), green (G), and blue (B).

Figure 3 shows the calculated colors corresponding to the transmitted light for silver blocks with various size parameters: width and height. Bright and vivid colors, which cover the entire visible color range, are represented by changing the structure parameters. Three colors indicated by black outlined boxes correspond to three representative colors: red, green, and blue. For the red color, when the silver block has the height (*H*) of 130 nm and the width (*W*) of 240 nm, the transmittance spectrum exhibits a peak at 670 nm and showed a good red color. The selected green color and blue color cases exhibit transmittance peaks at 510 and 470 nm, respectively, when their respective silver blocks have the (*H*, *W*) = (210 nm, 160 nm) and (170 nm, 280 nm) (Figure 3). 

**Figure 4:**
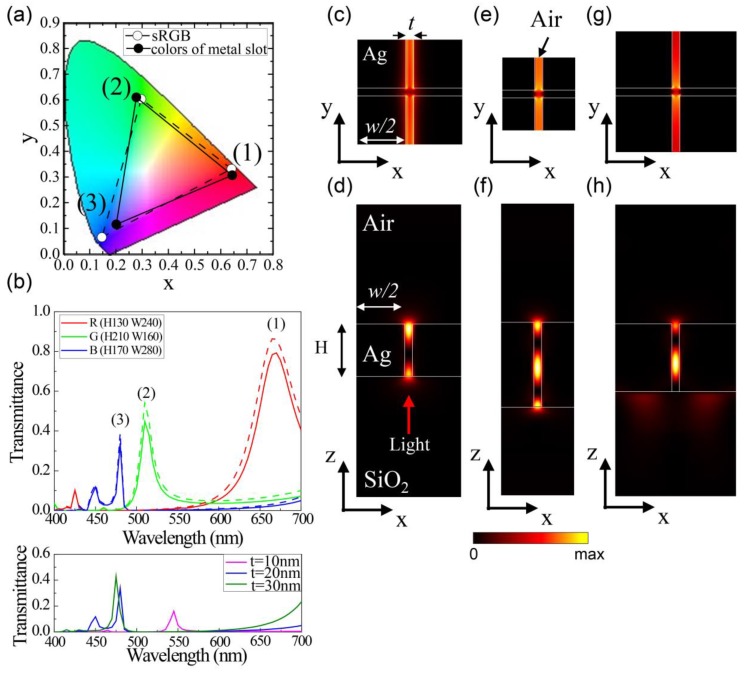
(**a**) Transmitted colors of silver blocks (solid lines) and sRGB area (dashed lines) in CIE color coordinate. (1), (2), and (3) indicate the three representative colors shown in Figure 3 and the structural height and width (*H*, *W*) of silver blocks for each RGB color are (130 nm, 240 nm), (210 nm, 160 nm), and (170 nm, 280 nm), respectively; (**b**) (Top) Transmittance spectra of three representative structures. When light is incident from SiO_2_ substrate (air) side, transmittance spectra are indicated as solid (dashed) lines. (Bottom) Transmittance spectra of blue structure for different slot thickness of 10, 20, and 30 nm; (**c**–**h**) Electric field intensity profiles of the transmitted light when linearly polarized light *E*_x_ with a wavelength at the transmission peak is normally incident from SiO_2_ substrate for three-color structures; (**c**,**e**,**g**) show top-views of transmitted light measured from air side; (**d**,**f**,**h**) show cross-sectional views; (**c**–**h**) correspond to (1), (2), and (3).

The three representative transmitted colors shown in Figure 3 are quantitatively located in the CIE color space, in which red, green, and blue are indicated by (1), (2), and (3) in Figure 4a. The respective transmittance spectra are plotted in Figure 4b. In Figure 4a, the transmitted color space of the silver blocks, which is represented by solid lines linked by (1), (2), and (3), covers almost 90% of sRGB area, indicated by dashed lines. For the three colors, red, green, and blue, the peak transmittances were estimated to be 0.79, 0.45, and 0.34, respectively, at each peak position (Figure 4b). On the other hand, when light is incident from the air side, the transmittance spectra are indicated as dashed lines in Figure 4b. Peak wavelengths and overall spectral shapes of the transmittance curves are same with them of the incidence from the SiO_2_ substrate side (solid lines), except for the slightly large peak transmittance. However, if the circumference of the silver blocks is filled with SiO_2_, a large increase of the refractive index from 1.0 to 1.5 induces a large red-shift of the transmittance peak. For example, the peak wavelength of green structure shifts from 510 to 670 nm.

In order to investigate the effect of the slot thickness, we calculated the transmittance curves for the different thickness of 10 nm (magenta), 20 nm (blue), and 30 nm (olive) for the blue structure, as shown in the bottom of Figure 4b. As the slot thickness decreases, the transmission peak shifts to the longer wavelengths because of the larger effective index of the metal–insulator–metal plasmonic mode [34,35]. On the other hand, the transmittance increases for the smaller slot thickness in the blue structure. Since the transmission peak depends on the air slot size sensitively, the fluctuation of the gap size can induce the broadening of the transmission peak, thereby reducing the color gamut of the proposed structure.

Figure 4c–h show the electric field intensity mode profiles of RGB color structures. Figure 4c–h correspond to (1), (2), and (3) marked in Figure 4a, respectively. These modes could be analyzed as the plasmonic slot modes excited in the metal–insulator–metal waveguide in which high energy was confined in the air slot. These strong energy confinements were effectively acted upon by the high transmission of light.

Although the areal filling factors of the air slot were only 13.8%, 21.0%, and 12.9% for RGB structures, relatively large transmissions of 80%, 43%, and 38% were observed because of resonant tunneling with the plasmonic slot mode.

The plasmon modes of the proposed structure is basically constructed by the coupling of SPPs excited at two metal interfaces in the metal–insulator–metal waveguide [34,36]. Since the end faces of the air slot to free space and SiO_2_ substrate operate as two mirrors, Fabry–Perot (FP) plasmonic resonance can be supported [34,36]. Therefore, as the height (*H*) increases, the cavity length increases and the resonant wavelength red-shifts, as shown in Figure 2. Since smaller air slot thickness (t) induces larger effective index [34,35], the effective height of the Fabry–Perot plasmon resonance increases, resulting in red-shifts of the peak wavelength. As the width decreases and the ratio of the cross-connecting region to the whole air slot increases effectively, then the peak wavelength slightly shifts to short wavelength because the electric field intensity at the cross-connecting points are weak, as shown in Figure 4c,e,g.

## 4. Angular Distribution of Transmitted Light

**Figure 5:**
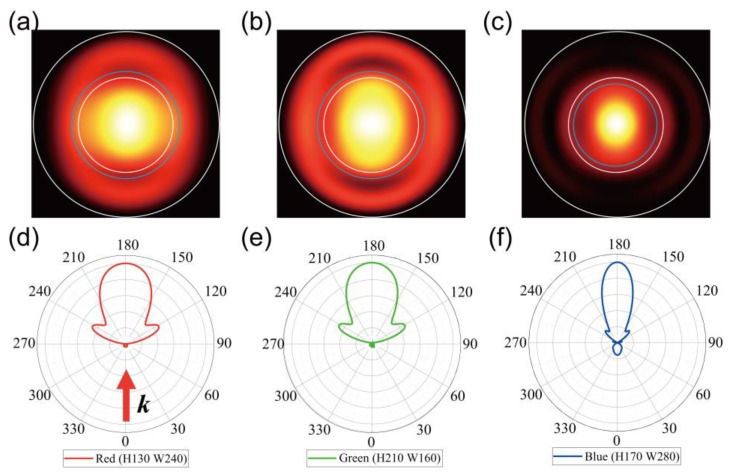
Far-field images of transmitted light for three representative structures with colors (**a**) red, (**b**) green, and (**c**) blue, described in Figure 3, for *Ex* polarized light. Here, the wavelengths of the incident light are 670, 510, and 480 nm, which are the peak wavelengths of the transmission spectra for three structures. The blue dashed circles indicate the boundary at which half of the total energy area is transmitted. The white solid line and white dotted circles indicate the boundary at 90° and 45° area from transmission direction, respectively. Polar graphs of the transmitted light for (**d**) red, (**e**) green, and (**f**) blue colored structures, respectively, obtained from far-field patterns of (**a**–**c**).

In order to investigate the angular distribution of the transmitted light, we calculated the far-field patterns of the transmitted light propagating into free space for three representative structures indicated in Figure 3. Figure 5 shows the far-field images of the structures with colors, red, green, and blue (Figure 5a–c), and the emission intensity polar graphs according to azimuthal angles (Figure 5d–f). The far-field image shows the strong directional emission of the transmitted light from *Ex* polarized light, in which most of the transmitted light is emitted at relatively narrow angles. Here, strong emission is observed in the center of the far-field patterns, corresponding to the transmission direction, normal to the surface of the silver blocks. In the case of red and green colors, half of the total emitted light, indicated by blue dashed circles in Figure 4a,b, is distributed within ±26° from the transmission direction, and in the case of blue, it is located within ±19° that is narrower than the red and green cases (Figure 5c). Next, we plotted the intensity distribution of the transmission light using the far-field profile data as a polar graph to grasp the more detailed characteristics of the transmitted light. The intensity value in each graph was normalized to the maximum value calculated from each data. The polar graphs shown in Figure 5d–f indicate the directional emission of the transmitted light into normal direction to the surface of silver blocks and smaller divergence angle of blue structure than those of red and green structures.

## 5. Conclusions

In this study, we proposed color filters that were constructed using silver blocks and cross-patterned air slots on a silica substrate. The silver blocks had two parameters of width (*W*) and height (*H*), and the thickness (*t*) of air slot was fixed to 20 nm. By using FDTD simulation, we calculated the transmission spectra for various structure parameters, which covered the entire visible color range, obtaining color data from the spectra.

When the silver blocks had width of 240 nm and height of 130 nm, the transmitted light showed red color, the spectrum peaked at 670 nm, and the transmittance was almost 0.8 of the incident light. The silver blocks also showed green and blue colors when the block had a specific parameter of width (*W*) and height (*H*). The green color was observed in the transmission when the silver block had a width of 160 nm and height of 210 nm. The peak of the green color case spectrum was positioned at 510 nm and its transmittance was 0.45. When the silver block had a width of 280 nm and height of 170 nm, a blue color was observed. The blue color case had the spectrum peak positioned at 480 nm and the transmittance was 0.34. These optimized colors from the proposed structure had 90% area of sRGB in the CIE color coordinate.

For the far-field patterns of the transmitted light, the structures with colors red, green, and blue showed divergence angles of ±26°, ±26°, and ±19°, respectively, from the transmission direction, showing relatively narrow emission angles.

Finally, we confirmed that the proposed structure could operate the color filters that cover 90% of sRGB area and has a narrow energy distribution of transmitted light. We expect that the proposed structure can apply color filters to nanoscale optical devices for filtering colors, and can be operated with sensors. However, the proposed structure has a relatively low transmittance in green and blue. Hence, in further study, if we use a material that has lower losses in visible light, instead of silver, high transmittance efficiency for green and blue colors can be expected. Small transmittances are commonly observed in the short wavelength region, as shown in Figure 2a and Figure 4b, because the material absorption and reflectance are considerably large in the shorter wavelength. In fact, the absorption, reflectance, and transmittance are quantitatively estimated as (0.095, 0.115, 0.79), (0.13, 0.42, 0.45), (0.29, 0.38, 0.33) for the red, green, and blue structures. In order to increase the transmittance in the blue and green structures, the shape of the air slot can be modified to suppress the reflectance [37] or aluminum or silver–aluminum alloy can be considered because of their low absorption in the short wavelength range instead of silver [38,39].
